# Optimization of split-ring resonator slots using levy-opposition-enhanced Newton Raphson method for high-gain UWB Vivaldi antenna design

**DOI:** 10.1038/s41598-026-41244-5

**Published:** 2026-02-27

**Authors:** Hüseyin Özmen, Davut Izci, Rizk M. Rizk-Allah, Serdar Ekinci, Mariana Dalarsson

**Affiliations:** 1 Engineering Faculty Electrical - Electronics Engineering, Dicle University, Diyarbakır, Turkey; 2 Department of Electrical Engineering, KTH Royal Institute of Technology, Stockholm, Sweden; 3 Department of Electrical and Electronics Engineering, Bursa Uludag University, 16059 Bursa, Turkey; 4 Applied Science Research Center, Applied Science Private University, Amman, 11931 Jordan; 5 Basic Engineering Science Department, Faculty of Engineering, Menoufia University, Shebin El-Kom, 32511 Egypt; 6 Department of Computer Engineering, Bitlis Eren University University, Bitlis, Turkey

**Keywords:** Newton–Raphson, Based optimization (NRBO), Lévy-opposition-enhanced NRBO, Split-ring resonator, Ultra-wideband (UWB) Vivaldi antenna, Optimization, Engineering, Mathematics and computing

## Abstract

This paper presents a novel hybrid metaheuristic optimization algorithm, termed Lévy-opposition-enhanced Newton–Raphson-based optimization (NRBO-LO), and its application to the design of a high-gain ultra-wideband (UWB) antipodal Vivaldi antenna. The proposed algorithm enhances the conventional Newton–Raphson-based optimizer by integrating random opposition learning to improve population diversity and Lévy flight–based guided learning to strengthen exploitation, thereby mitigating premature convergence. The effectiveness of NRBO-LO is first rigorously evaluated using a comprehensive set of unimodal, multimodal, and composite benchmark functions, where it demonstrates superior convergence accuracy, robustness, and stability compared to several well-established metaheuristic algorithms. Following algorithmic validation, NRBO-LO is employed within a MATLAB–CST co-simulation framework to optimize the geometrical parameters of split-ring resonator (SRR) slots embedded on both the radiating surface and ground plane of a compact antipodal Vivaldi antenna fabricated on a low-cost FR-4 substrate. The optimized antenna occupies an area of 40 × 40 mm^2^ and achieves a continuous impedance bandwidth covering the entire 3.1–10.6 GHz UWB range by reducing the lower cutoff frequency from 4.8 GHz to approximately 3 GHz. A maximum realized gain of 9.2 dB is obtained, representing a significant improvement over the reference design. Comprehensive electromagnetic analyses are performed to assess antenna performance in both the frequency and time domains. The proposed design exhibits directive radiation characteristics, an average radiation efficiency of approximately 75% with a peak efficiency of 91%, stable group delay, and high fidelity factors across multiple antenna orientations, indicating minimal signal distortion. The results confirm that the combined use of the NRBO-LO algorithm and metamaterial-inspired SRR slots provides an effective and practical approach for the optimization of compact, high-performance UWB Vivaldi antennas suitable for radar, microwave imaging, and wireless communication applications.

## Introduction

Ultra-wideband (UWB) antennas play a vital role in modern communication, sensing, and imaging systems owing to their ability to operate over a broad frequency range while supporting high data rates and low power consumption. Among the various UWB antenna configurations, Vivaldi antennas have received significant attention due to their inherently wide impedance bandwidth, end-fire radiation characteristics, and relatively high gain. These attributes make Vivaldi antennas particularly suitable for applications such as microwave imaging, ground-penetrating radar, wireless communication systems, and medical diagnostics. Nevertheless, conventional Vivaldi antenna designs often suffer from limitations related to insufficient gain and restricted lower cutoff frequencies, especially when compact geometries and low-cost substrates are employed. As a result, substantial research efforts have been devoted to enhancing the radiation performance of Vivaldi antennas while preserving their wideband characteristics.

Previous studies have demonstrated the effectiveness of UWB Vivaldi antennas in practical applications. For instance, Guruswamy et al.^1^ proposed a compact Vivaldi antenna incorporating directors to improve directivity for breast cancer detection. Qashlan et al.^2^ introduced a flexible and compact Vivaldi antenna array for microwave imaging systems, emphasizing portability and mechanical adaptability. These works highlight the capability of Vivaldi antennas to support high-resolution imaging and compact system integration. In medical imaging applications, the wide operational bandwidth and high-frequency capability of Vivaldi antennas enable improved spatial resolution and penetration depth. Danjuma et al.^3^ demonstrated the use of Vivaldi antenna arrays for breast cancer imaging, while Islam et al.^4^ developed a low-cost and portable UWB imaging system employing directional antennas to enhance tumor detectability. More recent studies have focused on gain enhancement and radiation control to further improve imaging accuracy. Özmen and Kurt^5^, for example, presented a gain-enhanced Vivaldi antenna specifically designed for breast phantom measurements, demonstrating improved tumor detection capability.

Beyond purely structural modifications, advances in signal processing and optimization techniques have contributed to the improvement of UWB imaging systems. Algorithms such as the delay multiply-and-sum (DMAS) method and its iterative variants have been shown to enhance image reconstruction quality and detection accuracy when combined with UWB antenna arrays^6,7^. These hybrid approaches underscore the importance of jointly optimizing antenna structures and associated computational techniques to achieve superior system-level performance.

In parallel with these developments, metamaterial-inspired structures have emerged as an effective means of enhancing antenna performance. Due to their ability to manipulate electromagnetic wave propagation, metamaterials have been widely employed to improve key antenna characteristics, including impedance bandwidth, gain, radiation efficiency, and directivity. Several studies have reported significant performance improvements by integrating metamaterial elements into Vivaldi antenna designs. Slimi et al.^8^ presented a metamaterial-based directional Vivaldi antenna achieving size reduction and gain enhancement for breast cancer detection. Islam et al.^9^ employed index-near-zero metasurfaces to enhance the bandwidth and gain of Vivaldi antennas in a microwave imaging system. Additional works have demonstrated the effectiveness of metamaterial lenses, double-negative structures, and index-near-zero metasurfaces in extending bandwidth and improving radiation performance across a wide frequency range^10–14^. Collectively, these studies confirm that metamaterial-inspired designs provide a powerful framework for tailoring the electromagnetic behavior of Vivaldi antennas.

Alongside structural enhancements, metaheuristic optimization algorithms have been extensively adopted for antenna parameter optimization due to their capability to handle nonlinear, multimodal, and highly constrained design spaces^15^. Among such approaches, the Newton–Raphson-based optimization (NRBO) has recently attracted attention owing to its simplicity and strong convergence characteristics^16^. However, like many population-based optimizers, conventional NRBO may suffer from premature convergence and limited global exploration when applied to complex electromagnetic optimization problems. To address these limitations, this study proposes an enhanced hybrid optimization algorithm, termed Levy-opposition-enhanced Newton–Raphson-based optimization (NRBO-LO). The proposed algorithm integrates two complementary strategies: random opposition learning (ROL)^17^, which improves population diversity and exploration capability, and Lévy flight–based guided learning (LGL)^18^, which enhances exploitation through adaptive step-size control. The performance of the proposed NRBO-LO algorithm is first rigorously validated using a comprehensive set of unimodal, multimodal, and composite benchmark functions, where it is shown to outperform several state-of-the-art metaheuristic optimizers in terms of accuracy, robustness, and convergence stability.

Following algorithmic validation, the NRBO-LO algorithm is applied to the electromagnetic design of a compact antipodal Vivaldi antenna intended for UWB radar and imaging applications. The antenna is implemented on a low-cost FR-4 substrate with overall dimensions of 40 × 40 mm2. To enhance both impedance bandwidth and radiation performance, a pair of symmetrical split-ring resonator (SRR) slots is introduced on the radiating surface and ground plane of the antenna. Unlike conventional approaches where SRRs are treated as separate resonant inclusions, the SRRs in this work are incorporated as optimized slots that directly modify the antenna’s current distribution and effective electrical length. The geometrical parameters of the SRR slots are optimized within a MATLAB–CST co-simulation framework using the proposed NRBO-LO algorithm, subject to constraints on impedance matching and gain maximization.

Comprehensive electromagnetic analyses are carried out to evaluate the optimized antenna performance. Frequency-domain results demonstrate that the proposed design achieves a continuous impedance bandwidth covering the entire 3.1–10.6 GHz UWB range, extending the lower cutoff frequency from 4.8 GHz (for the reference antenna) down to approximately 3 GHz. The optimized antenna attains a maximum realized gain of 9.2 dB, representing a significant improvement over the conventional design. In addition, radiation characteristics, directivity, and efficiency are thoroughly analyzed, revealing an average radiation efficiency of approximately 75% across the operating band and a peak efficiency of 91%. Time-domain performance is also investigated through group delay and fidelity factor analyses, confirming stable signal transmission with minimal distortion, which is essential for UWB radar and imaging applications.

The main contributions of this work can be summarized as follows:

1. Development of an enhanced hybrid optimization algorithm: A novel metaheuristic optimization algorithm is proposed by integrating NRBO with random opposition learning and Lévy flight–based guided learning. The effectiveness of the algorithm is validated through extensive benchmark function analyses, demonstrating superior convergence accuracy and robustness compared to several well-known optimizers.
2. Optimization-driven metamaterial-inspired antenna design: The proposed NRBO-LO algorithm is employed within a MATLAB–CST co-simulation framework to optimize the geometrical parameters of SRR slots embedded in a compact UWB Vivaldi antenna. The optimized design achieves full UWB coverage, significant gain enhancement, and improved radiation efficiency on a low-cost FR-4 substrate.
3. Comprehensive frequency- and time-domain performance evaluation: The antenna performance is rigorously assessed through impedance, gain, radiation pattern, efficiency, group delay, and fidelity factor analyses, confirming the suitability of the proposed design for UWB radar, imaging, and communication applications.

The remainder of this paper is organized as follows. Section “Basics of NRBO” introduces the fundamentals of the NRBO algorithm, including its initialization procedure, search stages, and trap avoidance strategy. Section “The proposed NRBO-LO algorithm” presents the proposed NRBO-LO algorithm, detailing the integration of the random opposition learning and Lévy flight–based guided learning strategies. Section "Evaluation of NRBO-LO on standard benchmark functions" evaluates the performance of NRBO-LO on a comprehensive set of unimodal, multimodal, and composite benchmark functions and provides comparative analyses with several state-of-the-art metaheuristic optimizers. Section "Antenna design" describes the design of the compact antipodal UWB Vivaldi antenna and the integration of split-ring resonator slots. section "optimization process and obtained results" details the optimization process and presents the obtained frequency- and time-domain results, followed by a comparative discussion with previously reported designs. Finally, Section "Discussion" concludes the paper by summarizing the main findings.

## Basics of NRBO

The NRBO is a new optimization approach designed to address optimization challenges by integrating Newton’s method with contemporary optimization strategies. Traditionally, Newton’s approach identifies function roots by repeatedly refining initial estimates using gradient information, and in such a way directing the search for solutions^16^. The NRBO expands upon this concept by presenting three fundamental principles for investigating solution spaces: it utilizes Newton–Raphson search rule (NRSR) using gradients to approach optimal points, integrates iterative modifications with random variations to maintain diversity and prevent premature convergence, and implements a trap avoidance strategy (TAS) to circumvent local optima.

From the perspective of the optimization problem, the objective is to identify the optimal function value while balancing exploration and exploitation characteristics. The optimization procedure seeks to determine the ideal collection of choice variables that provide the minimal value for the assessed fitness function. This issue configuration has many restrictions that delineate the allowable range for these variables, ensuring their values stay within defined limits that restrict the potential values of the decision variables. These restrictions are articulated as lower and upper limits for each variable (${Z}_{j}^{n}$) over all dimensions ($j=\mathrm{1,2},\dots ,Dim$). By imposing these constraints, the framework precludes solutions that may be unrealistic or unachievable in real-world scenarios, where specific parameters must conform to stringent, viable boundaries.

### Initialization phase and fitness assessment

Within this population-optimization framework, a set of agents (${N}_{P}$) is created and revised throughout each iteration ($Iter$) until the maximum iterations ($Ma{x}_{Iter}$) are attained, with each agent represented by a decision vector (${Y}_{j}^{n}$) of dimensions $Dim$. The solution vectors pertaining to agents are randomly allocated between the specified boundaries (${UB}^{j}$ and ${LB}^{j}$), computed as follows:

1 $$Y_{j}^{n} \left( {Iter = 0} \right) = LB^{j} + \left( {\left( {UB^{j} - LB^{j} } \right) \times rand} \right) , n = 1:Np, \;j = 1:Dim$$

For each search agent (${Y}_{n}$) within the population, the objective function ($fitness$) is assessed using studied problem and is defined as ($fit({Y}_{n})$). Subsequently, the agents are ordered according to their fitness values to determine the optimal agent (${Y}^{BEST}$) with the minimum fitness value ($min(fit({Y}_{n}$))) and the worst solution (${Y}^{WORST}$) with the maximum fitness value ($max(fit({Y}_{n}$))).

### Stage 1: employing the NRSR

The NRSR is employed to revise each agent’s location within the population by using gradient-based information to facilitate optimization. This rule employs a formula that computes updates based on relative fitness values, iteratively altering the agents’ placements. The update method incorporates a random perturbation factor ($\Delta Y$), which is proportionate to the disparity between the optimal agent and the current agent, hence augmenting the diversity of solutions.

For each agent, the NRSR formula employs two designated solution vectors, ${Y}_{1}$ and ${Y}_{2}$, in conjunction with a normally distributed random variable ($randn$), to calculate a weighted difference ($\Delta Y_{12}$) between these vectors. This approach enables updates according to the agents’ locations instead of their fitness values, potentially enhancing computational efficiency by reducing recalculations as shown below:

2 $$NRSR\left( {Y_{1} ,Y_{2} } \right) = randn \times \left( {\frac{{\left( {Y_{1} - Y_{2} } \right) \times \Delta Y_{12} }}{{2 \times \left( {Y_{1} + Y_{2} - 2 \times Y_{n} } \right)}}} \right)$$

where $\Delta Y_{12} { }$ denotes the weighted differences among designated solution vectors as shown below:

3 $$\Delta Y_{12} = rand \times \left| {Y_{1} - Y_{2} } \right|$$

The new solutions are produced by amalgamating three components, each subjected to random perturbations to promote diversified exploration. The principal components are ($Y_{1} ,{ }Y_{2} and{ }Y_{3}$), computed repeatedly using factors that include prior optimal solutions and stochastic elements. The location update for each agent ($Y_{n}^{j}$) at each iteration incorporates modifications based on weighted averages of ($Y_{1} ,{ }Y_{2} and{ }Y_{3}$) as shown below:

4 $$Y_{n}^{j} \left( {Iter + 1} \right) = Rd_{1} \times \left( {Rd_{1} \times Y_{1n}^{j} \left( {Iter} \right) + \left( {1 - Rd_{1} } \right) \times Y_{2n}^{j} \left( {Iter} \right)} \right) + \left( {\left( {1 - Rd_{1} } \right) \times Y_{3n}^{j} \left( {Iter} \right)} \right),n = 1:Np,j = 1:Dim$$

where $Rd_{1}$ is a stochastic variable ranging from 0 to 1; $Y_{1} \left( {Iter} \right),{ }Y_{2} \left( {Iter} \right)and{ }Y_{3} \left( {Iter} \right){ }$ are constructed using further random variations and factors that account for disparities between the best and worst search agents, in addition to the average of current locations as follows:

5 $$Y_{1n} \left( {Iter} \right) = Y_{n} \left( {Iter} \right) - NRSR\left( {Z_{w} ,Z_{v} } \right) + a{*}\left( {Y^{BEST} - Y_{n} \left( {Iter} \right)} \right) + b{*}\left( {Y_{R1} \left( {Iter} \right) - Y_{R2} \left( {Iter} \right)} \right)$$

6 $$Y_{2n} \left( {Iter} \right) = Y^{BEST} - NRSR\left( {Z_{w} ,Z_{v} } \right) + a*\left( {Y^{BEST} - Y_{n} \left( {Iter} \right)} \right) + b*\left( {Y_{R1} \left( {Iter} \right) - Y_{R2} \left( {Iter} \right)} \right)$$

7 $$Z_{w} = Rd_{2} {*}\left( {\left( {Mean\left( {Y_{n} - randn{*}NRSR\left( {Y^{WORST} ,Y^{BEST} } \right),Y_{n} } \right)} \right) + Rd_{2} {*}\Delta Y} \right){ }$$

8 $$Z_{v} = Rd_{2} {*}\left( {\left( {Mean\left( {Y_{n} - randn{*}NRSR\left( {Y^{WORST} ,Y^{BEST} } \right),Y_{n} } \right)} \right) - Rd_{2} {*}\Delta Y} \right){ }$$

9 $$Y_{3n} \left( {Iter} \right) = Y_{n} \left( {Iter} \right) + \left( {2{*}\frac{Iter}{{Max_{iter} }}} \right) - 1 + a{*}\left( {Y_{2n} \left( {Iter} \right) - Y_{1n} \left( {Iter} \right)} \right)$$

where ${Y}_{R1}\left(Iter\right)$ and ${Y}_{R2}\left(Iter\right)$ represent two distinct randomly selected vector agents from the population; $a$ and $b$ are arbitrary values ranging from 0 to 1. A random number inside the interval (0,1) is represented by $R{d}_{2}$.

### Stage 2: employing TAS

In the NRBO, the TAS is employed to assist agents in avoiding local optima, hence averting premature convergence. This strategy creates fresh locations by using the best-performing agent (${Z}^{BEST}$) and the current agent’s position (${Z}_{n}\left(Iter\right)$).

10 $$Y_{TAS} \left( {Iter} \right) = \left\{ {\begin{array}{*{20}c} {Y_{n} \left( {Iter + 1} \right) + XA\left( {Iter} \right) + XB\left( {Iter} \right), if\mu_{a} < 0.5} \\ {Y^{BEST} + XA\left( {Iter} \right) + XB\left( {IteR} \right), otherwise} \\ \end{array} } \right.$$

11 $$XA\left( {Iter} \right) = \theta_{1} \times \left( {\left( {\mu_{a} \times Y^{BEST} } \right) - \left( {\mu_{b} \times Y_{n} \left( {Iter} \right)} \right)} \right){ }$$

12 $$XB\left( {Iter} \right) = \left( {\frac{{\theta_{2} \times 2 \times Iter}}{{Max_{Iter} }} - \theta_{2} } \right) \times \left( {\left( {\mu_{a} \times Mean\left( {Y_{n} } \right)} \right) - \left( {\mu_{b} \times Y_{n} \left( {Iter} \right)} \right)} \right){ }$$

13 $$\mu_{a} = \left( {1 - \beta } \right) + 3 \times \beta \times Rd_{3}$$

14 $$\mu_{b} = \left( {1 - \beta } \right) + \beta \times Rd_{3} { }$$

15 $$\beta = \left\{ {\begin{array}{*{20}c} {1 if\;Rd_{3} > 0.5} \\ {0 otherwise} \\ \end{array} } \right.{ }$$

**Fig. 1:**
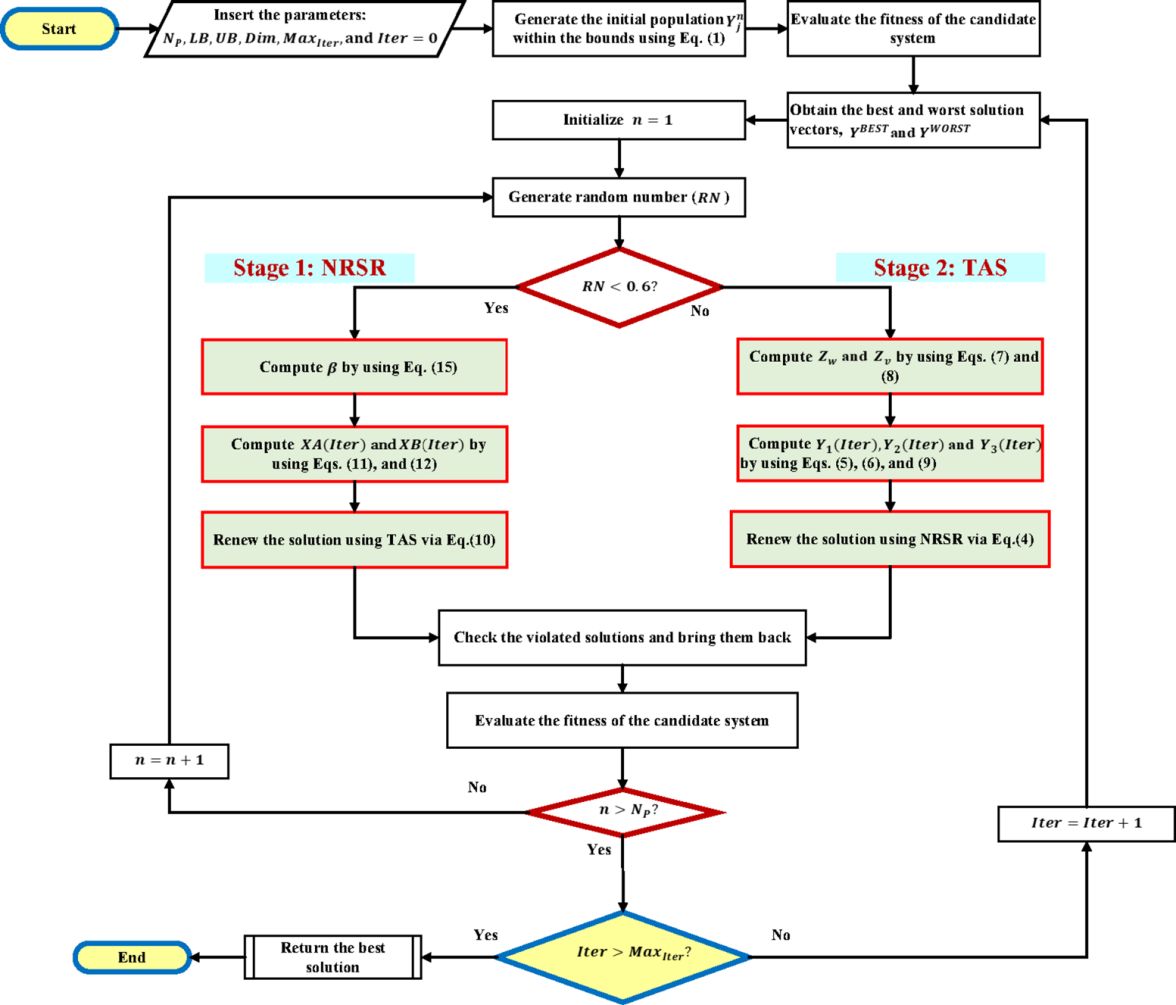
Flowchart of the NRBO.

In the NRBO, the TAS is implemented to assist agents in evading local optima, hence avoiding convergence stagnation dilemma. This strategy creates fresh locations by using the top-performing agent ($Y^{BEST}$) and the current agent’s position ($Y_{n} (Iter$)). The revised location, ${Y}_{TAS}$, is established by two scenarios contingent upon a parameter $\beta$. The new location is determined by modifying either the present agent’s position or the optimal agent’s position using two supplementary components, $XA(Iter)$ and $XB(Iter)$. These changes add diversity and are impacted by variables such as the optimal agent’s location, the population mean, and the current agent’s position, scaled by randomized parameters ${\theta }_{1}$ and ${\theta }_{2}$. The fundamental stages of the NRBO process are shown in Fig. 1, demonstrating the integration of TAS into the algorithm to improve overall diversification and solution quality. . The stages of the NRBO process may be succinctly stated as follows:

- *Step* 1: The NRBO parameters are defined (${N}_{P}, LB, UB, Dim, and Ma{x}_{Iter}$) together with the weighting factors (${P}_{1}and {P}_{2}$) in relation to the established objective model.
- *Step* 2: The initialization of population is performed using Eq. (1).
- *Step* 3: For each solution vector within the population, the fitness function is assessed using the candidate system to ascertain the performance of each agent in the optimization process.
- *Step* 5: The agents are rated according to their fitness values, with the agent with the lowest fitness value designated as the best agent and the one with the greatest fitness value classified as the worst agent.
- *Step* 6: A random number ($RN$) is created to facilitate the transition between the NRSR application via Step 6.a or the TAS stage via Step 6.b.

- *Step* 6.a: The NRSR is used to refine the agents’ placements using gradient-based information as delineated in Eq. (4). This phase facilitates the pursuit of the best solution by modifying the agents’ placements.
- *Step* 6.b: The TAS is executed using Eq. (10) to assist the agents in evading local optima and to promote exploration of the solution space.
- *Step* 7: Each solution vector is allocated the decision variables.
- *Step* 8: For each designated decision variables, the fitness function is assessed using the studied system to ascertain the performance of each agent in the optimization process.
- *Step* 9: The NRBO persists in iterating through the NRSR and TAS phases by repeated steps 5–8 until the maximum iteration limit is reached. Upon reaching this threshold, the algorithm ceases operation. The ultimate location of the best agent signifies the solution to the optimization issue.

## The proposed NRBO-LO algorithm

Due to its simplicity and robust exploration capabilities, NRBO is used to tackle real-world issues. The original NRBO consists of two search stages, both directed by the best search agent: the NRSR, which utilizes a gradient-based mechanism to facilitate the search process, and the TAS, which inhibits the algorithm from being trapped in local optima. The most effective search agent offers superior direction throughout the execution of the NRSR and TAS. Consequently, it is imperative that the best search agent be positioned at the global optimum. However, it is challenging to determine if the best search agent resides in optima due to insufficient a priori knowledge. If the best search agent reaches a local optimum, the population of agents will gather in that location, leading to a loss of population diversity eventually converging to a local optimum. To address these weaknesses, the conventional NRBO algorithm implements two strategies: random opposition learning (ROL) strategy and Levy flight-based guided learning (LGL) strategy. The subsequent portion of Section "The proposed NRBO-LO algorithm" offers a comprehensive explanation of these two enhanced techniques.

### The ROL strategy

The fundamental idea of opposition-based learning (OBL) stems from the understanding of oppositionality. OBL was suggested by Tizhoosh^19^. By integrating oppositeness, the solution area is partitioned into two distinct zones, hence facilitating a more efficient exploration of the solution space. OBL may augment the algorithm’s search space, including a wider array of possible optimum solutions, hence facilitating the discovery of superior solutions. It may also render the search procedure more focused and varied, hence enhancing the search speed. OBL evaluates both the optimum solution and its antithesis inside the search space, hence augmenting variety in the search and assisting in the avoidance of local optima. OBL is a novel approach in the field of intelligent optimization that may improve algorithm efficacy. It is extensively used in many optimization problems, including parameter tweaking, function optimization, and the training of machine learning models.

#### Definition 1

**(Opposite Number)**

^20^**:** Given a number $Y \in \left[ {LB,{ }UB} \right]$, its opposite number, represented as $\tilde{Y}$, is determined using the Eq. (16) as follows:

16 $$\tilde{Y} = LB + { }UB - Y$$

The concept of opposing numbers is applicable to higher-dimensional spaces as follows: 

#### Definition 2

**(Opposite Point)**

^15^**:** Let $Y = \left( {Y_{1} ,Y_{2} , \ldots ,Y_{Dim} } \right)$ be a point, where $Y_{1} ,Y_{2} , \ldots ,Y_{Dim} \in {\mathbb{R}}, Y_{j} \in \left[ {LB_{j} , UB_{j} } \right]$ for $j = 1,2, \ldots ,Dim$. The opposite point, denoted as $\tilde{Y} = \left( {\tilde{Y}_{1} ,\tilde{Y}_{2} , \ldots ,\tilde{Y}_{Dim} } \right)$, is calculated using Eq. (17) as follows.

17 $$\tilde{Y}_{j} = LB_{j} + UB_{j} - Y_{j}$$

Motivated by this concept, we propose an improved opposition strategy named Random Opposition Learning (ROL), aimed at ensuring accessibility to all areas of the search space. This method improves the exploration phase by including randomness, so preventing the algorithm from rigidly sticking to a deterministic trajectory. By emphasizing flexibility, the random term allows the algorithm to dynamically adjust and enhance its capacity to locate optimum solutions. The suggested ROL may be articulated as follows:

18 $$\tilde{Y}_{j} = rand \times \left( {LB_{j} + UB_{j} } \right) - Y_{j}$$

Here $rand$ is a random number that guarantees the newly generated positions do not repeat deterministically, thereby increasing the algorithm’s exploratory potential.

### The LGL strategy

Levy flight (LF) is a strategy grounded on the principles of random walk theory. LF imitates the stochastic flight patterns of animals in nature throughout the foraging process, facilitating enhanced leaps and variety. The importance of LF within metaheuristic algorithms is in its ability to improve search variety and global exploration capabilities. It facilitates detecting of optimum solutions with more efficiency and precision, particularly in complicated, high-dimensional, and irregular search areas. The step lengths may be determined probabilistically to navigate a novel search area. An approach for generating Lévy stabilization processes is provided in^21^. The Magenta technique employs the LF to produce stochastic numbers according to the formula:

19 $$Levy\left( \delta \right) = 0.05 \times \frac{x}{{\left| y \right|^{1/\delta } }}$$

where $x$ and $y$ define two stochastic variables described by a normal distribution, with $\sigma_{x}$ and $\sigma_{y}$ representing their respective standard deviations, as shown below:

20 $$x = N\left( {0,\sigma_{x}^{2} } \right),{ }y = N\left( {0,\sigma_{y}^{2} } \right){ }$$

The formula for $\sigma_{x} { }$ is expressed as follows:

21 $$\sigma_{x} = \left\{ {\frac{{{\Gamma }\left( {1 + \beta } \right)\sin \left( {\frac{\pi \beta }{2}} \right)}}{{\beta .\Gamma \left( {\frac{1 + \beta }{2}} \right).{ }2^{{\frac{{\left( {\beta - 1} \right)}}{2}}} }}{ }} \right\}^{1/\beta } ,{ }\sigma_{y} = 1$$

22 $$\Gamma \left( {1 + \beta } \right) = \mathop \smallint \limits_{0}^{\infty } t^{\delta } .{ }e^{ - t} { }dt$$

where $\Gamma$ is Gamma function; $\beta$ is a constant set to 1.5. Therefore, the proposed LGL combining the strengths of Levy flight idea through guided strategy to improve the suggested LGL may be articulated as follows:

23 $$Y_{{\text{LGL }}} = Y^{BEST} + Levy\left( \delta \right) \times \left( {Y\left( {Iter} \right) - Y^{BEST} } \right)$$

**Fig. 2:**
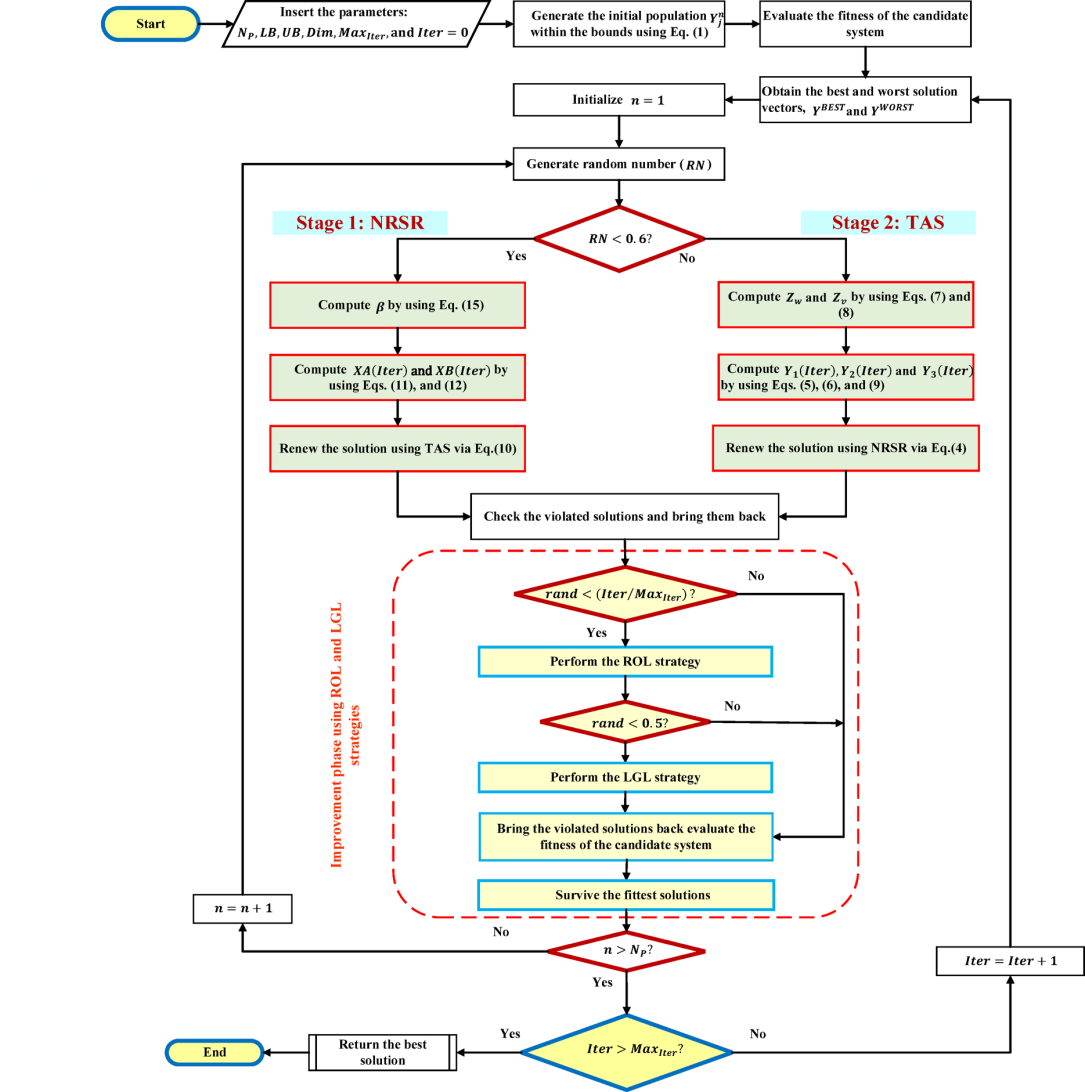
Flowchart of the proposed NRBO-LO.

In Eq. (23), the term $\left( {Y\left( {Iter} \right) - Y^{BEST} } \right)$ pulls the current position towards the best-known solution, encouraging exploitation of the promising region. On the other hand, the perturbation generated using a Levy flight distribution enriches the diversity of solutions, allowing the algorithm to escape local optima and explore other regions of the search space. Therefore, leveraging the Levy flight idea, the suggested optimization method may improve its exploration capabilities while preserving a balance with exploitation via the integration of small and large step sizes. The working of the improvement strategies is provided in Algorithm 1, while Fig. 2 illustrates the flowchart of the suggested NRBO-LO algorithm.

## Evaluation of NRBO-LO on standard benchmark functions

The performance of the proposed NRBO-LO was validated on a widely used set of classical benchmark functions^22^. These test functions, denoted as F1–F13 and F22–F23, represent unimodal, multimodal, and composite categories, enabling a comprehensive evaluation of both exploitation and exploration capabilities^23^. Functions F14–F21 were intentionally excluded from the analysis because their low dimensionality produced identical convergence behaviors across algorithms, which would not contribute meaningful comparative insights^23^. To establish a fair assessment, NRBO-LO was compared against several state-of-the-art optimizers, namely the grey wolf optimizer (GWO)^24^, sine cosine algorithm (SCA)^25^, gradient-based optimizer (GBO)^26^, jellyfish search (JS)^27^, Harris hawks optimization (HHO)^28^, and Tasmanian devil optimization (TDO)^29^, as well as the conventional NRBO^16^. The outcomes of this comparison are reported in Table 1, which provides the minimum, mean, maximum, and standard deviation values obtained over multiple independent runs.

**Table 1:** Comparative statistical evaluation of the NRBO-LO on standard benchmark functions.

Function	Metrics	GWO	SCA	GBO	JS	HHO	TDO	NRBO	NRBO-LO
F1	Minimum	1,25535E−16	0,001,845	4,2994E−137	1,89564E−19	4,3482E−119	1,50205E−91	0	0
	Mean	2,27479E−15	3,011,235	1,3804E−127	1,34368E−18	7,8557E−101	5,34034E−90	3,6854E−299	0
	Maximum	1,14714E−14	46,99,905	4,1301E−126	8,03762E−18	2,3305E−99	3,18929E−89	1,0748E−297	0
	St. dev	2,47135E−15	8,513,685	7,5398E−127	1,50625E-18	4,2533E-100	7,47408E-90	0	0
F2	Minimum	2,86681E−10	3,52E−06	1,57981E−70	4,10299E−11	3,81969E−62	9,20835E−48	9,1848E−159	1,3829E−239
	Mean	1,08058E−09	0,008,724	4,32229E−66	1,92564E−10	1,51566E−52	9,29793E−47	7,159E−151	9,8824E−217
	Maximum	3,84841E−09	0,059,904	3,97055E−65	6,90511E−10	4,22424E−51	7,32345E−46	2,0897E−149	2,9568E−215
	St. dev	6,962E−10	0,013,829	1,00046E−65	1,62059E−10	7,7042E−52	1,36738E−46	3,8119E−150	0
F3	Minimum	0,000,362,068	517,9381	1,1179E−116	0,00,361,177	2,4212E−101	1,25107E−28	3,424E−294	0
	Mean	0,045,247,331	6841,85	2,1314E−105	0,043,663,296	1,2665E−71	6,91394E−24	7,1998E−275	0
	Maximum	0,278,863,516	17,187,22	4,7781E−104	0,154,188,861	3,79949E−70	1,68983E−22	2,1599E−273	0
	St. dev	0,079,544,496	4785,358	8,8872E−105	0,035,256,989	6,93689E−71	3,08452E−23	0	0
F4	Minimum	0,000,157,965	10,08,083	1,61799E−63	8,86287E−08	7,99722E−59	5,1742E−39	3,7422E−154	7,731E−236
	Mean	0,000,927,047	27,52,885	6,21684E−59	2,26054E−07	2,6498E−50	2,00466E−38	2,1749E−147	6,317E−208
	Maximum	0,004,360,988	59,90,233	1,67611E−57	5,07158E−07	7,64657E−49	4,8862E−38	5,1074E−146	1,8113E−206
	St. dev	0,000,916,812	11,4365	3,05166E−58	1,06369E−07	1,39465E−49	1,09183E−38	9,5364E−147	0
F5	Minimum	25,74,962,064	29,50,786	19,28,646,319	0,009,170,581	3,49583E−06	24,9,149,761	26,296,465	16,02,778,739
	Mean	26,9,899,906	19,944,19	21,05,364,657	1,761,557,722	0,004,454,035	25,82,253,839	27,48,719,978	18,01,119,682
	Maximum	27,98,139,406	182,519,8	24,05,518,488	25,3,926,198	0,023,568,469	26,6,376,539	28,83,931,387	20,0,124,968
	St. dev	0,681,439,343	45,774,31	1,378,409,764	4,770,875,004	0,005,540,638	0,466,057,546	0,771,434,366	0,927,173,633
F6	Minimum	0,000,234,121	3,601,674	1,04897E−09	0,000,185,245	2,5118E−08	2,76011E−05	0,901,931,529	1,04037E−19
	Mean	0,464,369,978	7,335,087	1,12758E−07	0,001,069,793	7,22086E−05	0,000,485,227	2,278,986,205	4,76254E−16
	Maximum	1,226,475,205	22,93,299	1,6092E−06	0,004,528,459	0,000,842,037	0,004,984,479	3,12,713,626	9,20861E−15
	St. dev	0,343,262,241	4,417,024	3,18747E−07	0,001,068,416	0,00,015,994	0,00,090,285	0,544,254,052	1,75724E−15
F7	Minimum	0,000,541,259	0,007,448	4,21959E−05	0,000,594,782	2,64635E−06	9,63595E−05	9,12373E−06	1,10223E−05
	Mean	0,00,248,894	0,057	0,000,605,621	0,001,555,628	0,000,118,918	0,000,446,225	0,000,195,551	0,000,194,993
	Maximum	0,006,219,588	0,253,745	0,002,335,978	0,002,721,616	0,000,470,331	0,000,871,564	0,000,941,159	0,000,640,847
	St. dev	0,001,444,836	0,048,733	0,000,467,061	0,000,560,823	0,000,113,458	0,000,216,306	0,000,215,405	0,000,171,247
F8	Minimum	− 4986,7672	− 4864,38	− 11,734,8617	− 9543,97,157	− 12,569,4866	− 6869,24,302	− 6224,76,595	− 12,569,4866
	Mean	− 4453,32,704	− 3886,31	− 9169,28,503	− 5966,07,327	− 12,569,3679	− 5929,91,815	− 5081,07,539	− 12,468,1373
	Maximum	− 4043,24,643	− 3316,56	− 7924,56,866	− 4809,64,054	− 12,569,0079	− 5267,11,576	− 3863,85,505	− 11,661,3403
	St. dev	228,5,437,014	296,8952	829,4,096,126	1026,972,036	0,127,150,375	413,5,886,068	644,4,540,464	210,586,012
F9	Minimum	3,86535E−12	0,014,968	0	2,108,606,704	0	0	0	0
	Mean	3,785,768,728	29,22,913	0	10,17,982,431	0	0	0	0
	Maximum	12,39,942,466	137,2908	0	19,18,920,534	0	0	0	0
	St. dev	3,425,895,748	40,11,218	0	4,652,316,393	0	0	0	0
F10	Minimum	19,96,619,861	0,015,656	4,44089E−16	7,21276E−11	4,44089E−16	3,9968E−15	4,44089E−16	4,44089E−16
	Mean	20,07,347,806	10,58,108	4,44089E−16	3,29462E−10	4,44089E−16	5,65474E−15	4,44089E−16	4,44089E−16
	Maximum	20,18,395,786	20,2765	4,44089E−16	6,70952E−10	4,44089E−16	7,54952E−15	4,44089E−16	4,44089E−16
	St. dev	0,051,757,553	9,751,256	0	1,72658E−10	0	1,8027E−15	0	0
F11	Minimum	3,21965E−15	0,255,936	0	0	0	0	0	0
	Mean	0,003,759,167	0,75,917	0	6,66134E−17	0	0	0	0
	Maximum	0,03,117,412	1,13,235	0	1,44329E−15	0	0	0	0
	St. dev	0,008,897,203	0,249,809	0	2,68171E−16	0	0	0	0
F12	Minimum	3,09138E−05	0,54,948	9,68439E−11	5,06926E−06	2,13425E−10	1,36868E−06	0,091,144,253	1,17139E−20
	Mean	0,028,362,497	142,216,5	0,003,455,637	5,55431E−05	2,44292E−06	5,5887E−06	0,185,278,792	4,31074E−18
	Maximum	0,067,680,074	4,199,026	0,103,669,032	0,000,505,132	1,35021E−05	1,64467E−05	0,329,272,376	3,56316E−17
	St. dev	0,014,332,777	766,257,7	0,018,927,289	9,55394E−05	3,59094E−06	4,08032E−06	0,059,783,408	8,78796E−18
F13	Minimum	0,000,395,825	2,211,225	2,95736E−09	1,76041E−05	1,25911E−07	0,000,510,698	1,366,552,653	9,31921E−19
	Mean	0,340,748,546	8986,846	0,00,987,813	0,014,614,086	3,59462E−05	0,061,213,151	1,979,449,953	0,051,467,532
	Maximum	0,937,489,481	113,542,3	0,054,778,811	0,128,331,651	0,000,300,206	0,231,189,338	2,880,195,085	0,29,511,148
	St. dev	0,209,872,983	25,392,89	0,016,633,616	0,02,576,994	5,67355E-05	0,067,614,439	0,40,180,801	0,075,051,328
F22	Minimum	− 10,4,027,381	− 9,19,776	− 10,4,029,406	− 10,4,029,406	− 10,1,408,819	− 10,4,029,406	− 10,4,029,406	− 10,4,029,406
	Mean	−9,33,899,244	−4,2499	−8,19,944,059	−10,4,029,406	−5,42,207,089	−10,4,029,406	−8,569,238	−10,4,029,406
	Maximum	−5,08,756,732	−0,90,515	−2,76,589,733	−10,4,029,405	−5,08,094,325	−10,4,029,404	−2,76,589,733	−10,4,029,406
	St. dev	2,159,091,758	2,133,753	2,775,725,841	1,43706E−08	1,276,688,035	2,78772E−08	2,846,282,151	1,62432E−09
F23	Minimum	− 10,5,360,213	− 9,5167	− 10,5,364,098	− 10,5,364,098	− 10,5,228,119	− 10,5,364,098	− 10,5,364,098	− 10,5,364,098
	Mean	−9,85,075,848	−4,67,827	−8,10,274,901	−10,5,364,098	−5,99,817,136	−10,5,364,098	−9,40,619,495	−10,5,364,098
	Maximum	−2,87,010,821	−0,94,786	−2,42,173,403	−10,5,364,098	−5,12,598,218	−10,5,364,098	−3,8,354,268	−10,5,364,098
	St. dev	2,11,016,897	1,363,763	2,871,471,883	1,31944E-15	1,981,071,479	1,04043E-09	2,076,213,576	1,77636E-15

The unimodal functions, designed to evaluate exploitation ability, contain a single global optimum without local traps. NRBO-LO demonstrated outstanding performance in this group, consistently reaching the global optimum with both minimum and mean fitness values reported as 0 in cases such as F1, F2, and F3. The standard deviation in these cases was also 0, highlighting the stability of the method across repeated trials. In contrast, competing algorithms such as SCA and GWO showed mean errors on the order of 10^−3^ to 10^3^, with significantly larger variances. Even the original NRBO, while superior to most competitors, reported small but nonzero mean values in some functions. These findings confirm that the enhanced exploitation ability of NRBO-LO allows it to converge with remarkable precision and consistency.

The multimodal functions pose greater challenges due to the abundance of local minima. Here, NRBO-LO again exhibited clear superiority. For F9, which is notoriously difficult due to its highly repetitive landscape, NRBO-LO reached the exact global minimum of 0, while GWO and SCA recorded mean values above 3.7 and 29.2, respectively. Similarly, for F10, NRBO-LO achieved 4.44 × 10⁻^1^⁶, whereas SCA converged only to about 10.58 and GWO to 20.07 on average. In F11–F13, which include more irregular landscapes, NRBO-LO consistently achieved near-zero results with negligible deviations, while other algorithms struggled to maintain stability. These results confirm that the inclusion of random opposition learning and Levy-guided exploration substantially improves the ability to escape local optima and converge toward the true global solution.

Composite functions represent the most complex benchmarks, as they combine features from both unimodal and multimodal functions. On F22, NRBO-LO converged to the theoretical optimum of − 10.4029, with zero variance across runs. Other algorithms, such as GWO and SCA, exhibited much poorer accuracy, with mean results of − 9.34 and − 4.25, respectively. A similar trend was observed for F23, where NRBO-LO consistently achieved − 10.5364, again with zero deviation, while competing methods deviated significantly from this value. These results highlight the robustness of NRBO-LO in handling highly irregular search spaces where most algorithms face difficulties.

## Antenna design

**Fig. 3:**
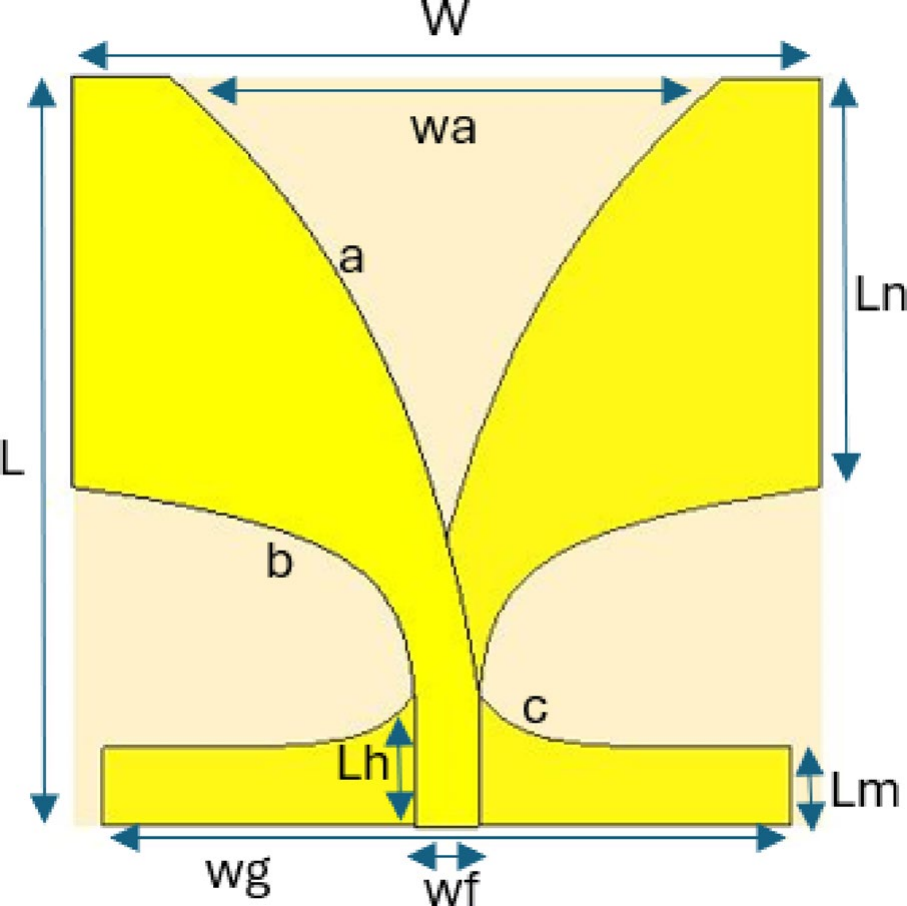
Vivaldi antenna before optimized SRR slots.

A high-gain antipodal Vivaldi antenna operating within the 3.1–10.6 GHz frequency range is designed using FR-4 substrate with a dielectric constant of 4.4, chosen for its low cost and easy availability. The antenna dimensions are 40 × 40 mm^2^ with a height of 1.6 mm, and simulations were conducted using CST Microwave Studio. Initially, a reference Vivaldi antenna was designed, where it was observed that the starting frequency remained at 4.8 GHz, and the maximum gain reached 7.7 dB at 9 GHz. The Vivaldi antenna presented in Fig. 3 and its geometrical parameters are presented in Table2. In Fig. 3a–c are the opening rates of the curves and that curves can be calculated using relevant formulas in^5^. To enhance both bandwidth and gain, split-ring resonator (SRR) slots were incorporated into the design. The geometrical parameters of the SRR structure were optimized using the NRBO-LO algorithm, developed in this study, to further improve antenna performance.

**Table 2:** Geometrical parameters of the Vivaldi antenna.

Parameter	W	L	wa	Ln	Lm	Lh	wf	wg	a	b	c
Value(mm)	40	40	29.6	21.9	4.17	7	3.48	36.84	0.06	0.38	0.48

## Optimization process and obtained results

**Fig. 4:**
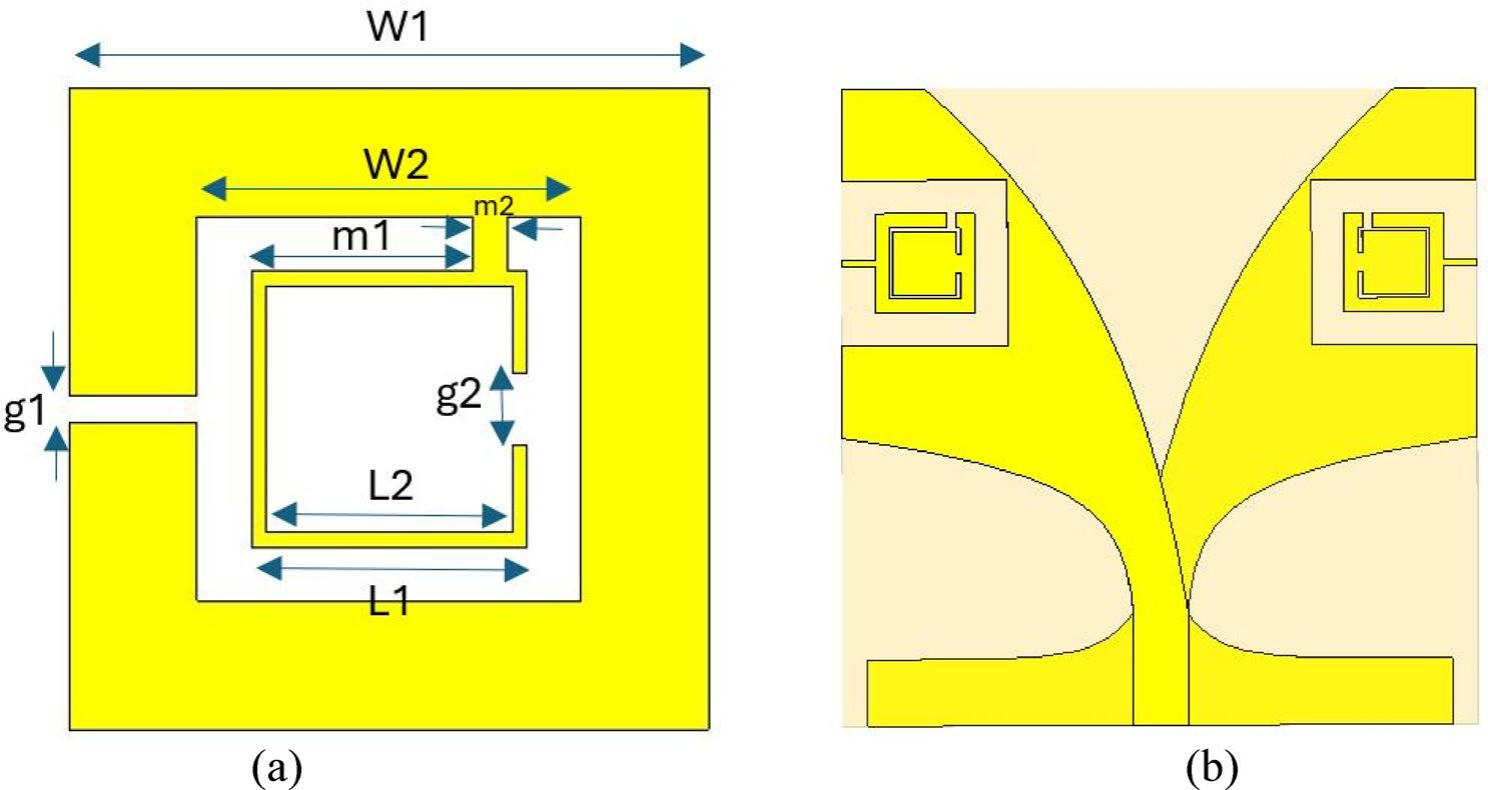
SRR (**a**) and the Vivaldi antenna with SRR slot pair **b**).

In this study, the enhancement of the antenna performance is pursued by integrating two identical and symmetrical square-shaped split ring resonator (SRR) slots into the antipodal Vivaldi antenna structure. These SRR slots are introduced by removing corresponding regions from both the antenna’s radiating surface and the ground plane. Unlike conventional approaches where slots might be analyzed separately, in this work, the SRR slots are modeled and optimized as integral parts of the antenna structure, ensuring an overall and realistic evaluation of their impact on antenna behavior. SRR structure and the Vivaldi antenna with SRR slot pair are shown in Fig. 4a, b respectively:

The geometric parameters of the SRR slots, consisting of eight distinct variables as depicted in the design schematics, are systematically optimized with the objective of improving key antenna characteristics such as bandwidth and realized gain. The optimization is carried out using a hybrid Newton–Raphson-Based Optimization algorithm enhanced with Levy-Opposition (NRBO-LO), which is implemented within the MATLAB environment. Meanwhile, the full-wave electromagnetic simulations required to evaluate antenna performance, including reflection coefficient (S_11_) and gain, are performed using CST Microwave Studio. MATLAB and CST operate in a synchronized co-simulation framework, enabling iterative exchange of design parameters and simulation results.

At the initial stage of the optimization, a 20 × 8 population matrix is randomly generated in MATLAB, representing 20 individuals with 8 parameters each, which are used to determine the 8 geometric lengths of the SRR that illustrated in Fig. 4a. These parameter sets are transmitted to CST, where antenna simulations are executed, and the resulting S_11_ and gain values are then fed back to MATLAB. The optimization objective is formulated as the maximization of gain, subject to the critical constraint that the average reflection coefficient S_11_ across the frequency range remains below -10 dB, as expressed by:

24 $${\text{Cost function}} = {\mathrm{max}}\left( {{\mathrm{gain}}} \right)$$

Subject to the following constraint:

25 $$\frac{1}{N}\mathop \sum \limits_{n = 1}^{N} S_{11} (f_{i} ) \le - 10 dB$$

Here, gain is the antenna’s realized gain at the desired frequencies, S_11_ is the reflection coefficient at each frequency point f_i_ within the frequency range and N is the total number of frequency points in the considered range. This constraint guarantees acceptable impedance matching and minimal reflection over the desired bandwidth. The optimization process verifies this constraint at each iteration. Should the constraint be violated, the NRBO-LO algorithm updates the SRR parameters accordingly, and the new parameters are sent back to CST for subsequent simulation. This feedback loop continues iteratively until either the constraint is met or the maximum number of iterations, set to 50, is reached.

To enforce the S_11_ constraint effectively, a penalty method is incorporated within the fitness evaluation. If the average S_11_ exceeds  - 10 dB, a penalty term is applied to discourage the selection of non-compliant solutions, thereby steering the optimization towards antenna designs that satisfy both gain maximization and reflection coefficient requirements.

**Fig. 5:**
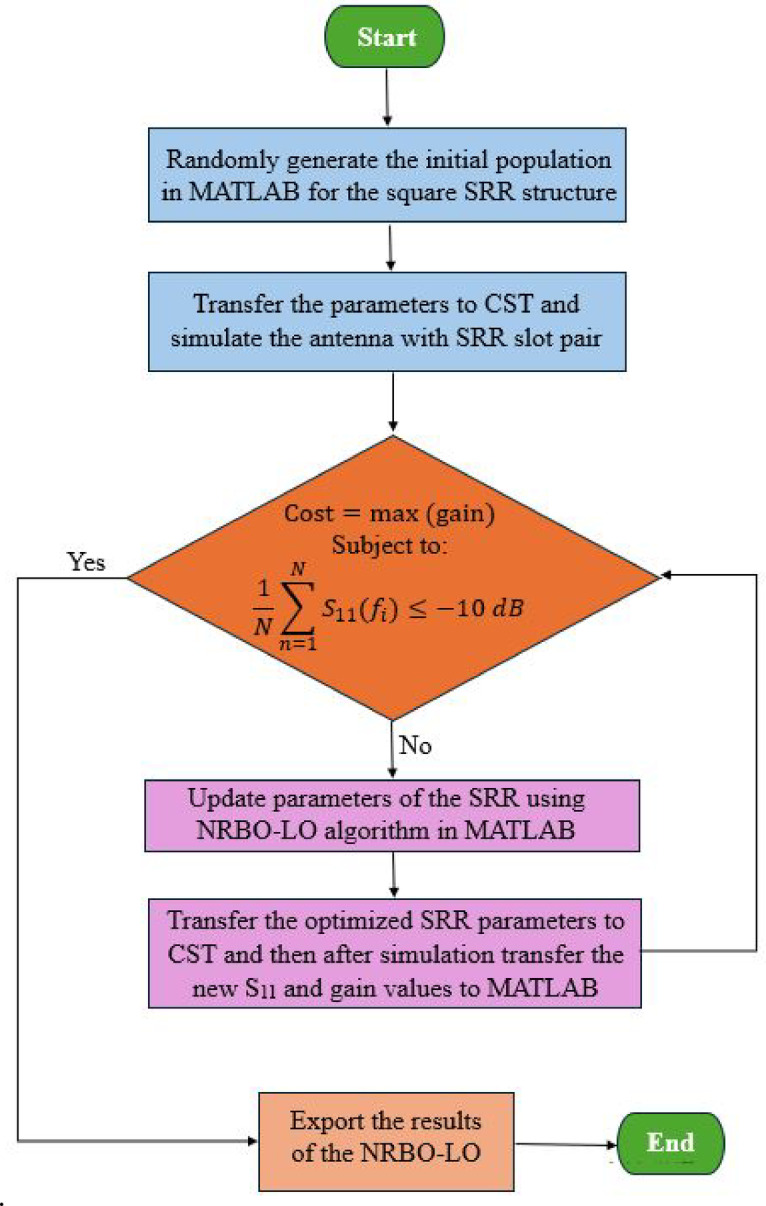
Optimization flowchart.

**Fig. 6:**
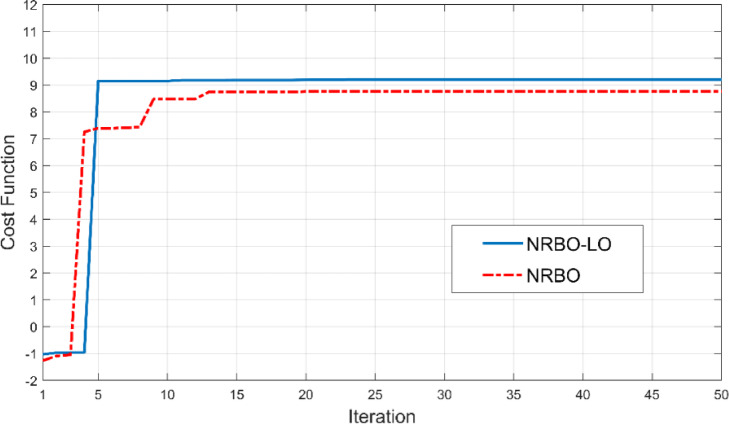
Cost function values versus iteration numbers.

Through this integrated MATLAB-CST co-simulation framework combined with the NRBO-LO algorithm, the study aims to achieve an optimized SRR slots loaded Vivaldi antenna configuration that exhibits enhanced radiation performance with respect to gain and bandwidth, while maintaining satisfactory impedance matching across the operating frequency band. The flowchart illustrating the NRBO-LO optimization process is presented in Fig. 5. The final geometrical parameters of the optimized SRR structure which is used as the slot on the radiating surface and ground surface are presented in Table 3. As a result of optimizing the structure in Fig. 4 as a slot on the front and back surfaces of the Vivaldi antenna using NRBO and NRBO-LO, the graph in Fig. 6 was obtained.

**Table 3:** Optimized parameters of the SRR.

Parameter	W1	W2	g1	L1	L2	g2	m1	m2
Value(mm)	10.4	6.24	0.4	4.48	4	1.2	3.6	0.56

**Fig. 7:**
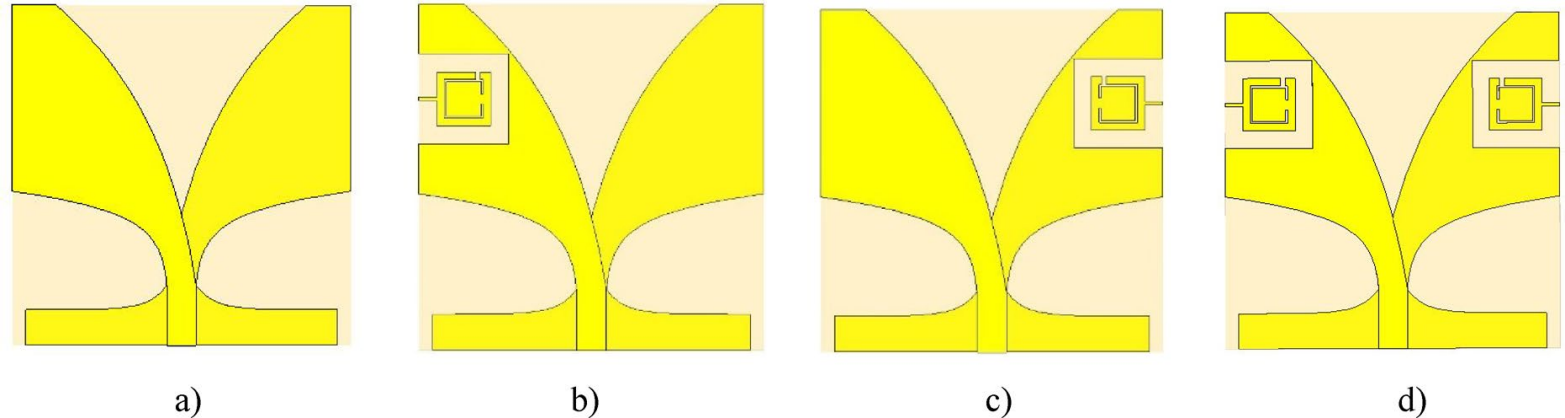
Different antenna modifications: (**a**) reference antenna, (**b**) SRR slot on the radiating surface, (**c**) SRR slot on the ground surface, (**d**) SRR slots on both radiating surface and ground surface.

According to the Fig. 6, when the SRR geometry is optimized using NRBO-LO, the gain rapidly reaches 9.2 dB, whereas when optimized using NRBO, it reaches a maximum of 8.76 dB at a slower rate. This graph demonstrates that the NRBO-LO algorithm is highly effective in improving bandwidth and antenna gain and performs better than NRBO. In order to see the effects of the added SRR’s at different positions on antenna surface, results of four different modification were investigated. These modifications are shown in Fig. 7.

**Fig. 8:**
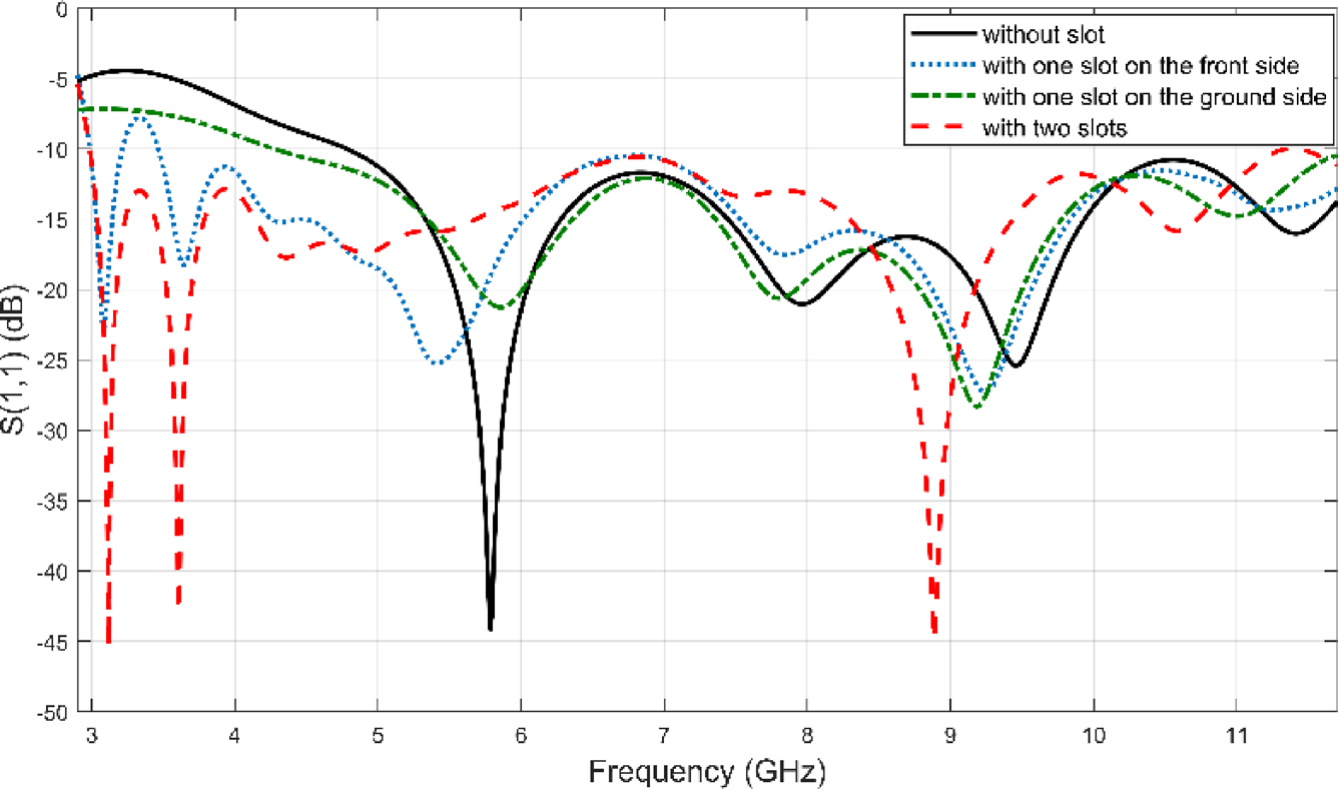
S_11_ values for different antenna modifications.

To conduct a comparative step-by-step performance analysis of the SRR integration, four distinct configurations were investigated, as shown in Fig. 7a–d. We initially designed the final symmetrical SRR structure (Fig. 7d) and optimized its 8 geometric parameters using the NRBO-LO algorithm. The resulting optimized SRR parameters (from Table 3) were then systematically applied to the simpler configurations, Fig. 7b, c, without re-optimization, to isolate the performance impact of the SRR’s placement and symmetry on the radiator and ground plane. The S_11_ simulation results of these modifications can be seen in Fig. 8. As can be seen from the Fig. 8, the reference Vivaldi antenna doesn’t cover the desired 3.1–10.6 GHz frequency range, as its operating bandwidth starts at 4.8 GHz. Introducing an SRR slot on the radiator side significantly enhances bandwidth performance; however, the S_11_​ value exceeds − 10 dB in the 3.2–3.5 GHz range, preventing full compliance with the target frequency band. When an SRR slot is applied only to the ground plane, the lower cutoff frequency improves to 4.2 GHz compared to the reference design. However, the most effective improvement is achieved in the fourth configuration, where SRR slots are symmetrically implemented on both the radiator and ground plane. Thus, they significantly modify the current distribution and effective electrical length of the antenna. As a direct result, the operating band is shifted from 4.8 GHz (for the conventional Vivaldi antenna without slots) down to 3 GHz in the proposed design. Thereby fully covering the intended 3.1–10.6 GHz range. These results highlight the crucial role of SRR structures in bandwidth enhancement, demonstrating that a symmetrical slot arrangement yields the most optimal antenna performance.

The results confirm that integrating SRR slots significantly influences impedance matching and bandwidth enhancement. The symmetrical placement of SRR slots on both the radiator and ground provides the most extensive bandwidth improvement, making the antenna well-suited for UWB applications. The present study demonstrates that SRR-based design modifications are effective in optimizing the performance of Vivaldi antennas, particularly in achieving wideband operation while maintaining low reflection coefficients across the intended frequency range.

**Fig. 9:**
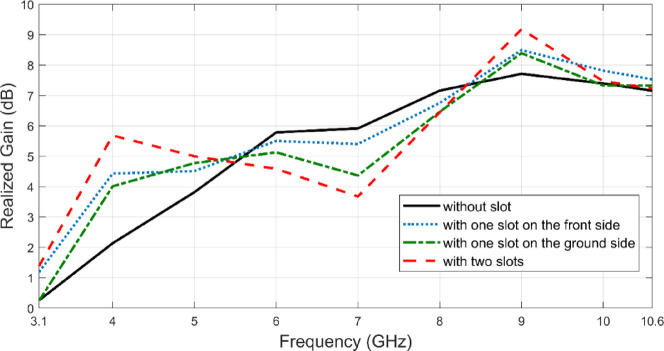
Realized gain values for different antenna modifications.

According to Fig. 9, the reference antenna achieves a maximum realized gain of 7.7 dB at 9 GHz. When an SRR slot is introduced only on the radiator side, the maximum gain increases to 8.5 dB, while placing the SRR slot solely on the ground plane results in a maximum gain of 8.4 dB. The most significant improvement is observed when SRR slots are symmetrically incorporated on both the radiator and ground plane, achieving a peak gain of 9.2 dB, the highest among all configurations. SRRs, effectively modify the current distribution on the antenna surface and increase radiation efficiency. The proposed pair of symmetrical SRR slots contributes to beam focusing and thus increased directivity by modifying the effective electrical length of the antenna and redirecting the surface currents. This increased directivity is the primary driver for the increase in gain. This is confirmed by the symmetrical SRR slotted configuration which increased the peak gain from 7.7 to 9.2 dB. Although there is a reduction in gain within the 5.5–8.5 GHz range compared to the reference antenna, the overall enhancement across the 3.1–10.6 GHz band, along with the increase in maximum gain from 7.7 to 9.2 dB, demonstrates that utilizing SRR as a slot not only contributes to bandwidth expansion but also plays a crucial role in improving antenna gain performance.

**Fig. 10:**
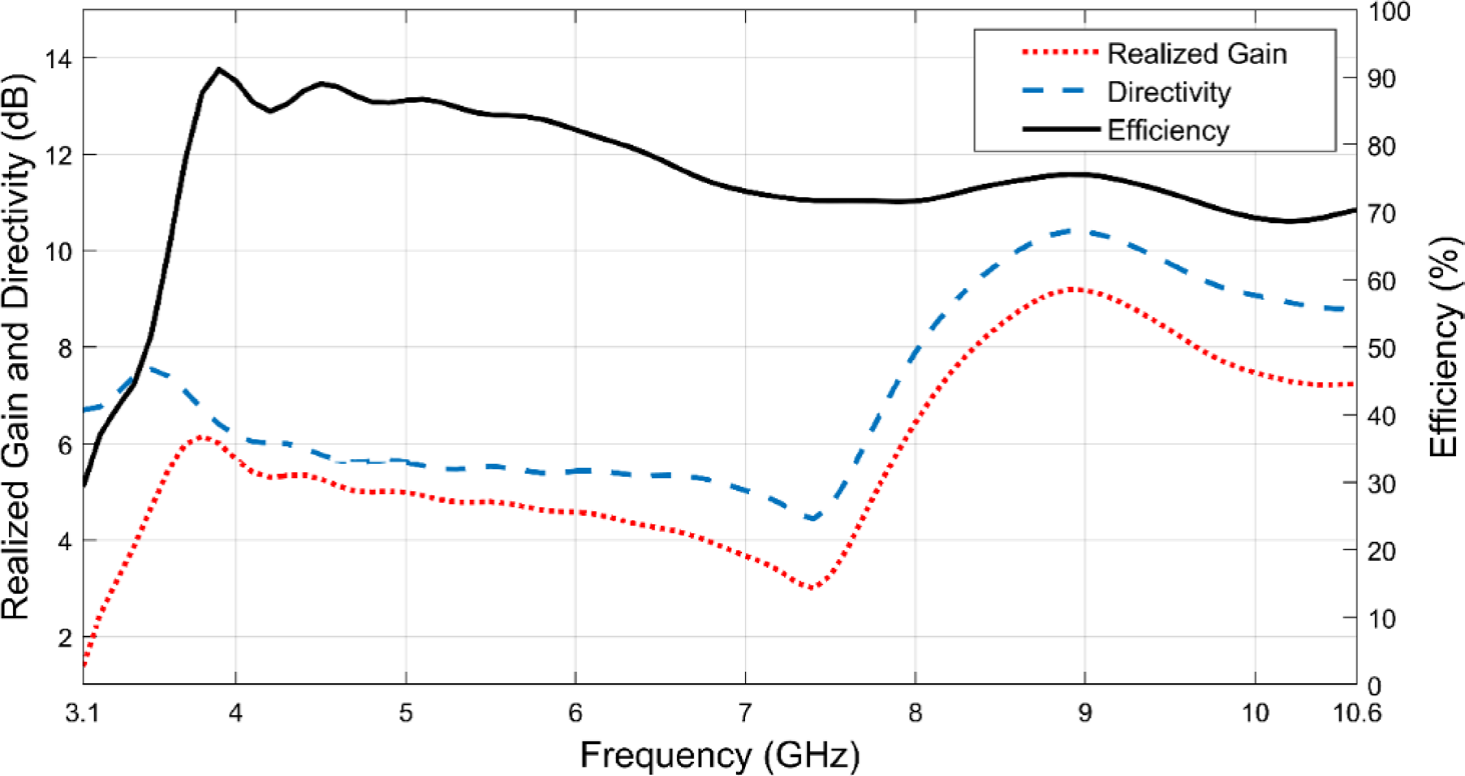
Directivity and efficiency of the proposed antenna.

In Fig. 10 it seems that at 8.9 GHz, the proposed Vivaldi antenna achieves a directivity of 10.4 dB, and its radiation efficiency can be assessed through the ratio of realized gain to directivity. A high-efficiency antenna effectively radiates most of the input power, whereas low-efficiency designs suffer from losses due to impedance mismatches and material absorption. The proposed antenna demonstrates a peak efficiency of 91% at 3.9 GHz, indicating minimal power loss at this frequency. Across the entire operating frequency range (3.1–10.6 GHz), the antenna maintains an average efficiency of 75%, which is a reasonable performance for directive ultra-wideband (UWB) applications. Although there may be frequency-dependent variations in efficiency, the overall performance suggests that the antenna effectively converts input power into radiated energy.

**Fig. 11:**
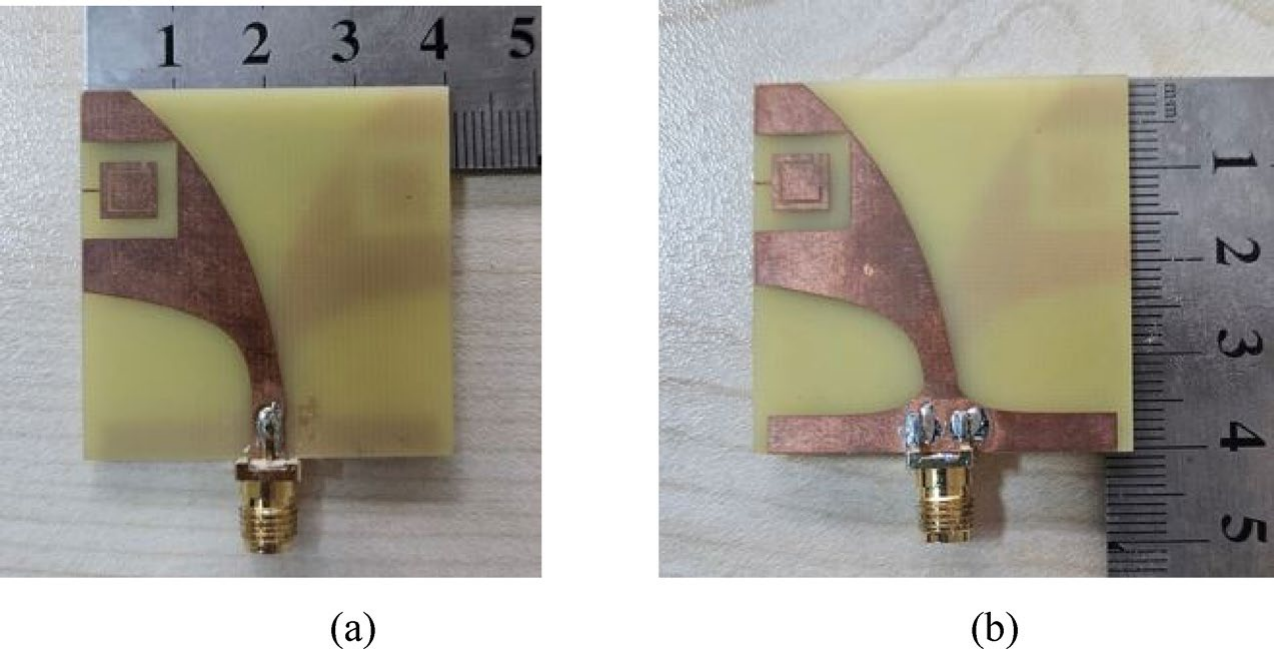
Fabricated antenna: (**a**) front side, (**b**) back side.

To experimentally validate the simulated S_11_ performance and radiation characteristics, the proposed antenna was fabricated using PCB technology. The front and back views of the fabricated antenna are presented in Fig. 11a, b, respectively.

**Fig. 12:**
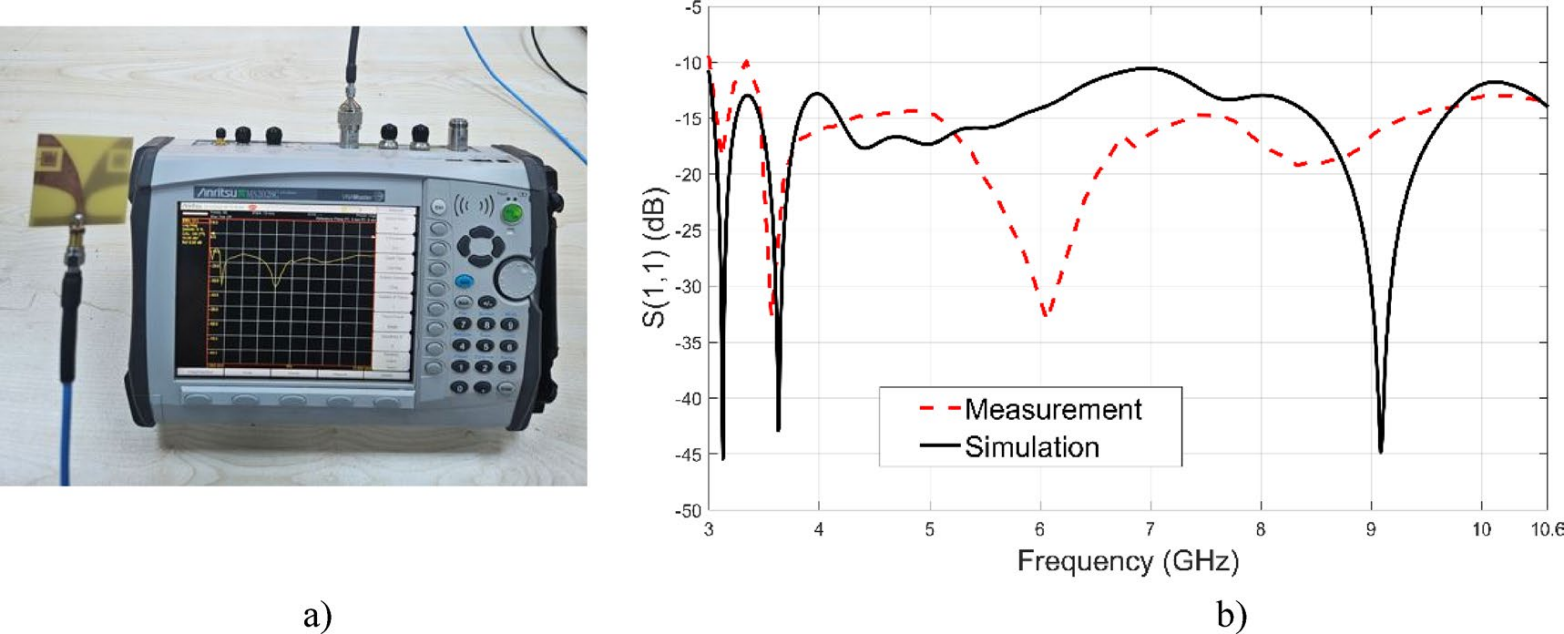
(**a**) S_11_ measurement setup, (**b**) Simulated and measured S_11_ of the proposed antenna.

Figure 12 illustrates the experimental validation of the proposed antenna. As shown in Fig. 12a, the fabricated antenna is measured using a vector network analyzer (VNA) to obtain the reflection coefficient. Figure 12b presents a comparison between the simulated and measured S_11_ results as a function of frequency.

A good agreement is observed between the simulation and measurement over the operating band, with slight discrepancies due to fabrication tolerances, connector losses, and measurement uncertainties. The results confirm the validity of the proposed antenna design and its predicted performance.

**Fig. 13:**
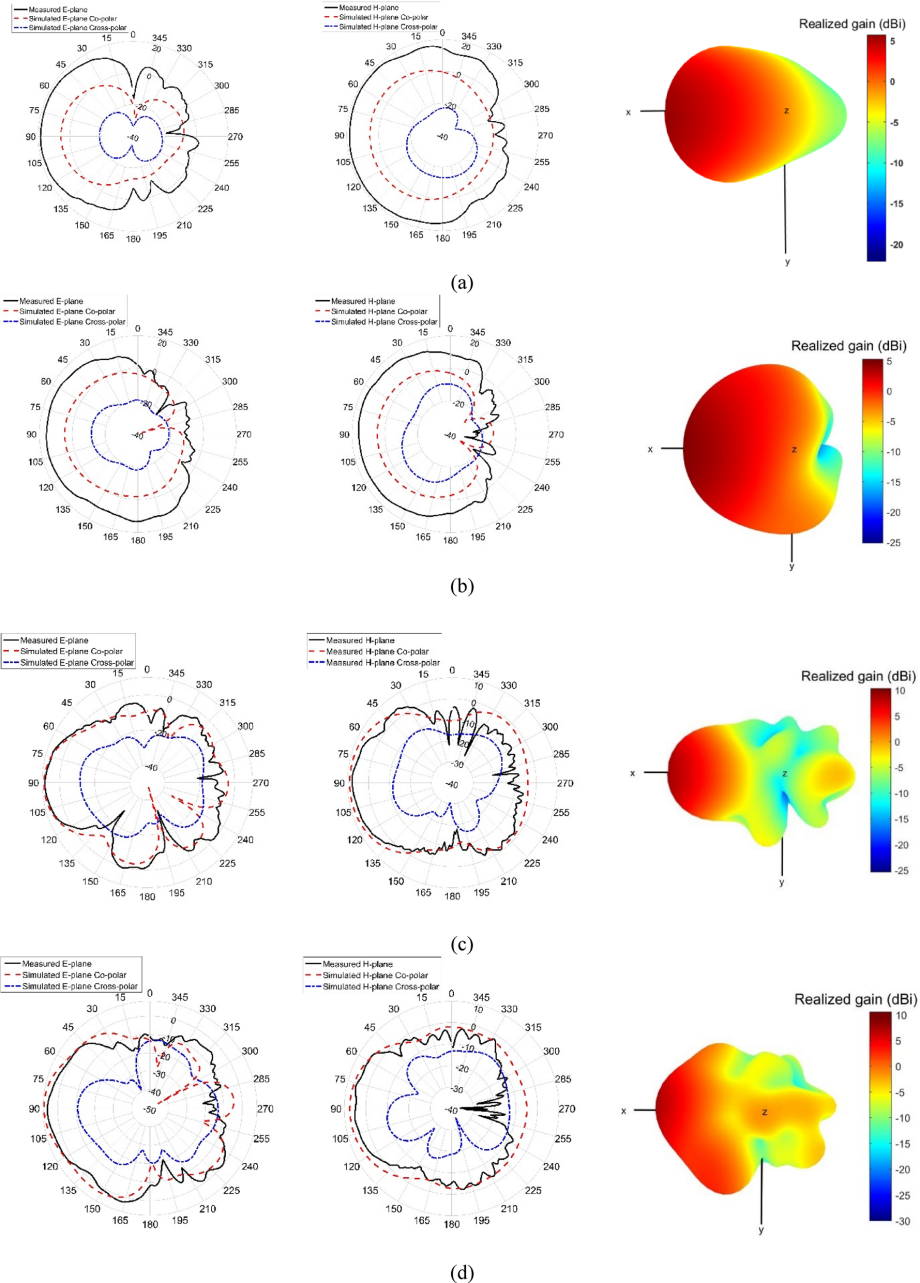
E-plane and H-plane co-polarization and cross-polarization patterns, together with the 3D radiation patterns of the designed antenna at (**a**) 4 GHz, (**b**) 6 GHz, (**c**) 9 GHz, and (**d**) 10.5 GHz.

In UWB radar applications, the radiation characteristics of an antenna, including its directivity and beam concentration, play a crucial role in ensuring effective energy transmission toward the target. To achieve this, the radiation pattern of the proposed Vivaldi antenna was analyzed in CST Microwave Studio and further validated through experimental measurements. The polar E-plane and three-dimensional radiation patterns of the antenna at 4 GHz, 6 GHz, 9 GHz, and 10.5 GHz are presented in Fig. 13, where both simulated and measured results are shown. The results indicate that the antenna exhibits a highly directive radiation pattern, with good agreement between simulation and measurement, confirming its suitability for UWB radar applications by efficiently focusing energy in the desired direction.

**Fig. 14:**
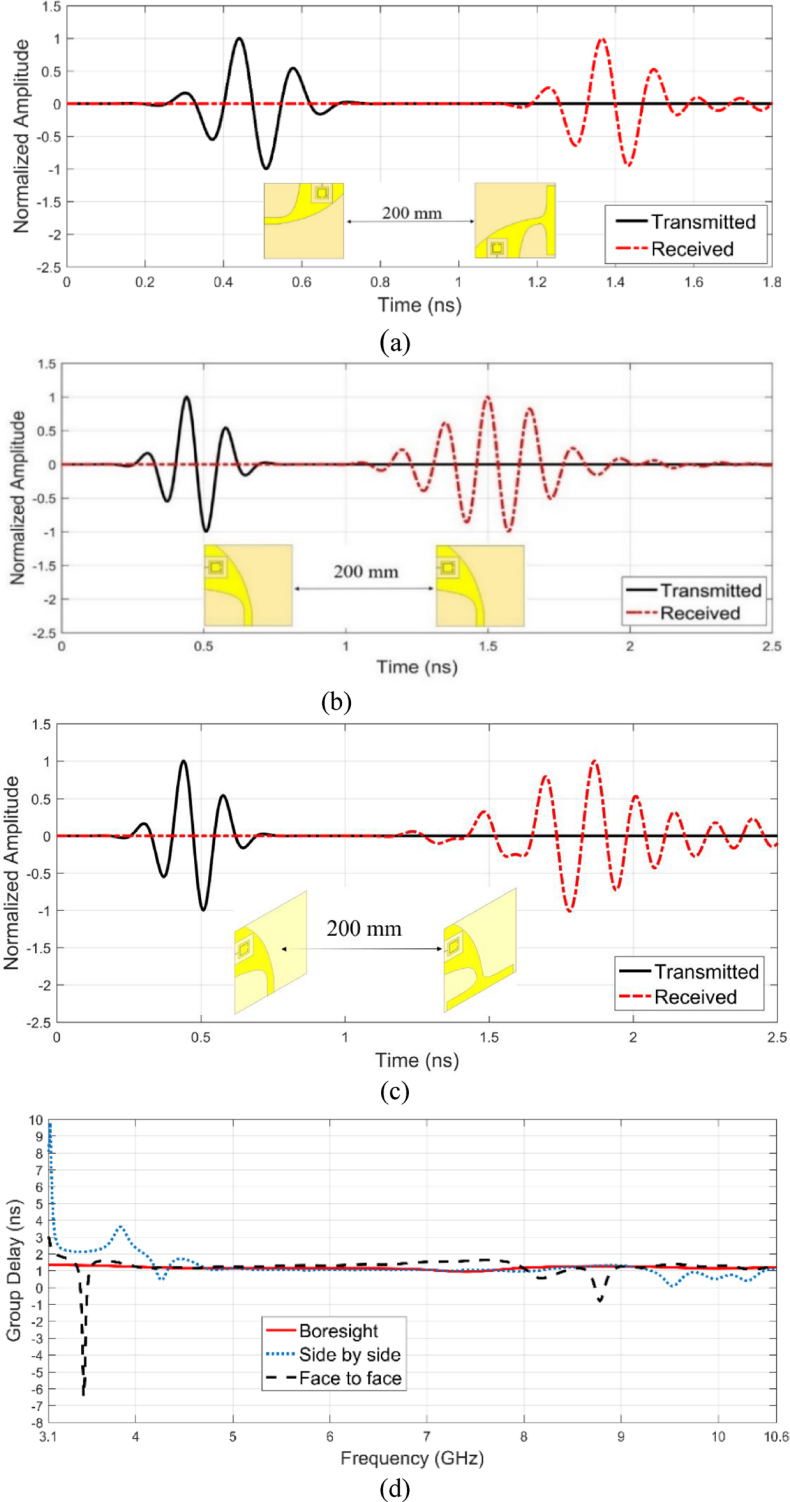
Simulated waveforms in three different antenna orientations: (**a**)boresight, (**b**) side by side, (**c**) face to face and (**d**) group delay.

Figure 14 presents the simulated time-domain waveforms for the proposed Vivaldi antenna across three distinct orientations: boresight (a), side-by-side (b), and face-to-face (c). Additionally, the figure includes the corresponding simulated group delay (d), which reflects the phase linearity and signal distortion across the operational bandwidth.

For the proposed Vivaldi antenna, the minimum group delay is 0.94 ns, while the maximum group delay is 1.35 ns and average group delay is 1.17 ns within the 3.1–10.6 GHz band in the boresight orientation. These values indicate that the antenna maintains a relatively stable group delay across the operational bandwidth, minimizing phase distortion and ensuring reliable pulse transmission in the boresight configuration. The low variation in group delay suggests that the antenna exhibits good time-domain performance, which is essential for UWB applications such as high-data-rate communication, radar, and imaging systems. The obtained results demonstrate that the proposed design effectively reduces signal dispersion, making it suitable for applications requiring high-fidelity signal transmission with minimal waveform distortion.

A widely used metric for quantifying the similarity between the transmitted and received signals in time-domain systems, which indicates the level of signal distortion introduced by the antenna, is the fidelity factor. It is defined as the normalized cross-correlation between the transmitted waveform S_T_(t) and the received waveform S_r_(t), and can be calculated using the following equation:

26 $$FF = max_{{\tau^{d} }} \left| {\frac{{\mathop \smallint \nolimits_{ - \infty }^{\infty } S_{T} \left( t \right)S_{R} \left( {t - \tau^{d} } \right)dt}}{{\sqrt {\mathop \smallint \nolimits_{ - \infty }^{\infty } \left| {S_{T} \left( t \right)} \right|^{2} dt{*}\mathop \smallint \nolimits_{ - \infty }^{\infty } \left| {S_{R} \left( t \right)} \right|^{2} dt} }}} \right|$$

where $ma{x}_{{\tau }^{d}}$ is the time delay that maximizes the correlation. In this study, the fidelity factor was calculated based on the transmitted and received waveforms in the boresight, side by side and face to face antenna configurations shown in Fig. 12, resulting in a value of 97%, 89% and 79% respectively. This high fidelity factor in the boresight configuration indicates minimal distortion and excellent signal integrity throughout the UWB frequency band, confirming the antenna’s suitability for time-domain applications.

## Discussion

In this section, a comparison between the proposed antenna and several Vivaldi antennas reported in the literature is provided in Table 4. As seen, the proposed design achieves a wide impedance bandwidth of 3–11.7 GHz while maintaining a compact size of 40 × 40 mm^2^ on a low-cost FR-4 substrate, demonstrating a favorable balance between size, bandwidth, and gain. Compared to earlier works such as^1^, which operates over a similar UWB frequency range (3.1–10.6 GHz) but occupies a larger area of 48 × 45.7 mm^2^ with a lower maximum gain of 8.25 dB, the proposed antenna provides both size reduction and a noticeable gain improvement. Designs reported in^3^ and^4^ also exhibit larger physical dimensions (57 × 41 mm^2^ and 51 × 42 mm^2^, respectively) while achieving lower or comparable gain levels, highlighting the compactness and enhanced radiation performance of the proposed structure. Although some designs, such as^7^ and^9^, report slightly higher maximum gains (10 dB and 9.3 dB, respectively), these improvements come at the expense of significantly larger antenna sizes (e.g., 161.56 × 136.68 mm^2^ in^7^ and 77.72 × 60 mm^2^ in^9^) and, in some cases, the use of higher-cost substrates such as RT5880. In contrast, the proposed antenna achieves a competitive maximum gain of 9.2 dB using a substantially smaller size and a cost-effective FR-4 substrate, making it more suitable for compact and low-cost UWB systems. Furthermore, antennas reported in^8,10,30^ and^32^ exhibit either narrower operational bandwidths or considerably lower gain values, despite comparable or larger physical dimensions. The proposed antenna outperforms these designs by simultaneously offering wide UWB coverage, higher gain, and compact size. Compared to designs with extended bandwidths, such as^31^, the proposed antenna maintains a significantly smaller size while achieving comparable gain performance.

**Table 4:** Comparison of the antenna simulation results to similar works.

References	Frequency (GHz)	Size (mm^2^)	Max gain (dB)	Substrate
^1^	3.1–10.6	48 × 45.7	8.25	FR-4
^2^	3.8–4.8 and 9–10	25 × 20	2.24 and 2.7	Polyamide
^3^	3–9	57 × 41	N/R	FR-4
^4^	2.8–7	51 × 42	7.5	FR-4
^5^	3–10.6	36 × 36	8.5	FR-4
^6^	2.5–11	40 × 40	7.2	FR-4
^7^	1.1–10	161.56 × 136.68	10	FR-4
^8^	4.6–8	67 × 46	5.8	Roger 3010
^9^	2.7–11.2	77.72 × 60	9.3	RT5880
^10^	2–10.45	46 × 40	7.9	RT5880
^30^	4–16	50 × 25	3.53	FR-4
^31^	2.3–20	114 × 70	8.2	RT5880
^32^	1.63–8	50 × 50	9.03	FR-4
^33^	2.82–11	60 × 90	8.84	FR-4
^34^	3.02–12	29.4 × 32.2	7.3	FR-4
Proposed	3–11.7	40 × 40	9.2	FR-4

From the comparison, it is evident that the proposed design achieves a wide operating frequency range of 3–11.7 GHz, which is broader than many other antennas in the literature. In particular, the lower cut-off frequency is reduced compared to similar FR-4 based antennas, demonstrating the effectiveness of the metamaterial-inspired slots in extending the impedance bandwidth.

In terms of gain, the proposed antenna achieves a maximum realized gain of 9.2 dB, which is higher than most FR-4 based UWB antennas and comparable to antennas designed using high-performance substrates such as RT5880. This is a significant achievement, considering that FR-4 is a low-cost, lossy substrate, typically associated with reduced radiation efficiency. The improvement in gain can be attributed to the incorporation of the SRR slots, which enhance radiation efficiency by modifying the surface current distribution and effectively suppressing unwanted resonances.

The antenna also exhibits a compact size of 40 × 40 mm^2^, making it smaller than many wideband designs, while still maintaining good performance. Although some works^2,34^ report smaller geometries, their bandwidth and gain are considerably lower compared to the proposed antenna, limiting their applicability in broadband high-gain systems.

In summary, the proposed antenna offers an attractive balance of wide impedance bandwidth, high gain, compact size, and cost-effective fabrication on FR-4, outperforming many previously reported designs. These results confirm that the integration of the SRR slots into the Vivaldi structure provides an effective approach to enhance both bandwidth and radiation characteristics, making the antenna suitable for UWB wireless communication, radar, and sensing applications.

In this work, the primary contribution is the development and application of the novel hybrid Newton–Raphson-Based Optimization with Lévy Opposition (NRBO–LO) algorithm. While the classical NRBO algorithm demonstrates strong local convergence properties, its performance can degrade when applied to highly nonlinear and multimodal optimization problems such as antenna design. By integrating Lévy opposition-based learning, the proposed NRBO–LO algorithm improves global search capability and prevents premature convergence. This algorithm was employed to optimize the geometry of the SRR slots embedded on both the radiating arm and the ground plane of the Vivaldi antenna. The optimized slots successfully extended the operating bandwidth from 4.8 GHz (without slots) down to 3 GHz and enhanced the maximum realized gain to 9.2 dB. The results clearly demonstrate the effectiveness of the NRBO–LO algorithm in aiding metamaterial-inspired antenna design and underline its potential as a powerful tool for solving complex electromagnetic optimization problems.

## Conclusion

In this study, a novel hybrid metaheuristic optimization algorithm, referred to as NRBO-LO, was developed and successfully applied to the design of a compact, high-gain UWB antipodal Vivaldi antenna. The proposed NRBO-LO algorithm enhances the conventional NRBO framework by integrating random opposition learning to improve population diversity and Lévy flight-based guided learning to strengthen exploitation, thereby alleviating premature convergence and improving global search capability. The effectiveness of NRBO-LO was first validated through extensive evaluations on standard unimodal, multimodal, and composite benchmark functions. The results demonstrated that NRBO-LO consistently outperformed several well-established metaheuristic algorithms in terms of convergence accuracy, robustness, and stability, often achieving the theoretical optima with negligible or zero variance across multiple independent runs. These findings confirm that the proposed hybridization strategy significantly enhances both exploration and exploitation capabilities compared to the conventional NRBO. Following algorithmic validation, NRBO-LO was employed within a MATLAB–CST co-simulation framework to optimize the geometrical parameters of SRR slots integrated into a compact antipodal Vivaldi antenna fabricated on a low-cost FR-4 substrate. By embedding a pair of symmetrical SRR slots on both the radiating surface and the ground plane, the effective electrical length and surface current distribution of the antenna were favorably modified. As a result, the lower cutoff frequency was reduced from 4.8 GHz to approximately 3 GHz, enabling full coverage of the 3.1–10.6 GHz UWB band. The optimized design achieved a maximum realized gain of 9.2 dB, representing a substantial improvement over the reference antenna, while maintaining a compact footprint of 40 × 40 mm^2^. Comprehensive electromagnetic analyses were conducted to assess the antenna performance in both the frequency and time domains. The proposed antenna exhibited directive radiation characteristics, an average radiation efficiency of approximately 75% with a peak efficiency of 91%, and stable impedance matching across the entire UWB range. Time-domain investigations further confirmed the suitability of the design for UWB applications, as evidenced by low group-delay variation and high fidelity factors in multiple antenna orientations, indicating minimal signal distortion and reliable pulse transmission.

## Data Availability

The dataset used and/or anaylzed during the current study available from the corresponding author on reasonable request.
